# Short-duration treatment for latent tuberculosis in migrants: VDOT monitoring in Manaus, AM.

**DOI:** 10.1590/0037-8682-0530-2023

**Published:** 2024-04-05

**Authors:** Yan Mathias Alves, Sonia Vivian de Jesuz, Thaís Zamboni Berra, Vania Maria Silva de Araújo, Ethel Leonor Noia Maciel, Ricardo Alexandre Arcêncio

**Affiliations:** 1 Escola de Enfermagem de Ribeirão Preto, Departamento de Enfermagem Materno-Infantil e Saúde Pública, Ribeirão Preto, SP, Brasil.; 2 Universidade Federal de Mato Grosso - Campus Sinop, Sinop, MT, Brasil.; 3 Rede Brasileira de Pesquisa em Tuberculose, Rio de Janeiro, RJ, Brasil.; 4 Ministério da Saúde, Secretaria de Vigilância em Saúde e Ambiente, Brasília, DF, Brasil.

Tuberculosis (TB) is an infectious disease that requires appropriate treatment to prevent complications and spread. The strategy to end TB, aligned with the Sustainable Development Goals, sets ambitious targets, including an 80% reduction in incidence and a 90% reduction in mortality, with a focus on high-priority populations, including migrants and refugees^1^. Through the Political Declaration of the United Nations General Assembly High-Level Meeting on Tuberculosis, the importance of prioritizing at-risk groups and other vulnerable individuals is recognized^1^.

The fight against TB remains a major public health challenge in Brazil. The health and social crises exacerbated by the COVID-19 pandemic have had a negative effect on access to diagnosis and treatment of the disease. Progress made in the years leading up to the pandemic has stalled or reversed, and the resumption of actions has fallen short of what is necessary^2^. During the first year of the pandemic, an estimated 10.1 million people developed TB worldwide, but only 5.8 million (57.4%) were diagnosed and reported, representing an 18% reduction compared with 2019 when 7.1 million cases out of the estimated 10 million (71%) were reported worldwide. In 2021, an estimate 10.6 million people developed TB, of which 6.4 million (60.4%) were reported, indicating a reduction in the global underreporting of TB cases^3^.

Migrants and refugees face barriers to accessing healthcare, including lack of documentation, lack of awareness of the available services, logistical challenges in accessing services, and linguistic, cultural, and transit difficulties. In addition, migrants from high TB-burden countries such as Venezuela, may develop active TB that is not diagnosed in the first few years after migration. These difficulties contribute to delays in the diagnosis and treatment, thereby increasing the risk of disease transmission within the community. Moreover, long-term treatment reduces treatment adherence^4^.

With the economic crisis in Venezuela coupled with rising unemployment, one of the world's largest human migrations has taken place, with Brazil being the fifth most popular destination country. As a border region with Venezuela, the northern region of Brazil has become a gateway, accommodating Venezuelans, and taking responsibility for their internalization. During this process, Manaus, the capital of the state of Amazonas, needed to develop a plan with specific healthcare actions for this migrant population, including a plan to manage TB in migrants^1^.

The difficulty of monitoring the treatment of migrants in transit and during the internalization process is evident, and this challenge was exacerbated during the COVID-19 pandemic^4^. Therefore, it became necessary to consider new strategies for remote monitoring of this population. In Brazil, short-course treatment (3HP), combining high doses of isoniazid (H) and rifapentine (P) administered once a week for 3 months, is currently used as a short-term preventive therapy. Although the duration of 3HP is half the duration of conventional treatment, which lasts for 6 months, the effectiveness at curing TB is similar^5^.

New approaches and alternative healthcare interventions have been sought to fill the gaps and healthcare voids that remain, especially due to the pandemic. In 2015, the World Health Organization (WHO) established a Global Digital Health Task Force for TB to support the development of health innovations to improve TB care, disease prevention, and break the chain of TB transmission^6^. In 2017, video-observed treatment (VOT) was endorsed as an alternative to directly observed treatment (DOT)^7^.

In this context, the Video-Based Tuberculosis Treatment Monitoring System (VDOT) emerged to monitor individuals undergoing directly observed treatment for TB and implement short-course treatment (3HP) for TB in international migrants in Manaus.

Monitoring interventions can help improve TB treatment outcomes compared with unsupervised interventions and self-administered (SA) treatments. Innovative approaches to enhance and optimize supervised treatment interventions should be explored, especially during the current public health crisis triggered by the COVID-19 pandemic^8^. In this regard, guided by a patient-centered approach for individuals diagnosed with TB, support for affected countries is recommended to ensure the effectiveness of TB treatment. Among the established strategies is the use of digital technologies for the sustainability of short-course DOT, whether through messages or technological resources such as 99DOT and VOT that alert individuals with a TB diagnosis to regularly access their medication kits^8^.

VOT allows individuals diagnosed with TB to use a smartphone to record videos of their daily medication intake, including during weekends, eliminating distance barriers, reducing travel costs to health facilities, and providing affected individuals with greater autonomy to choose when and where to take their medications^9^. Story et al^9^ also emphasizes that healthcare professionals can support a larger number of individuals, thereby increasing the efficiency of the healthcare system.

Considering previous studies, the pre-existing problems related to access to healthcare services, and the repercussions of the COVID-19 pandemic on the Brazilian Unified Health System (SUS), it is imperative to reconsider adapting services to develop disease control actions to prevent the further spread of TB. Several benefits are expected, including a reduction in treatment duration, allowing for faster and more effective disease control to prevent its spread within the community. Telemonitoring of TB treatment can ensure greater adherence among individuals with TB, especially the migrant population, increasing their chances of cure and reducing the risk of drug resistance.

The implementation of the short-course treatment (3HP) has the potential to benefit not only migrants but also the general population because TB is a communicable disease. Adequate treatment of latent TB among migrants may protect the entire community from the spread of TB. Furthermore, by ensuring the health of migrants, social inclusion and respect for their human rights are promoted, thus strengthening the social fabric of the city.

**FIGURE 1:**
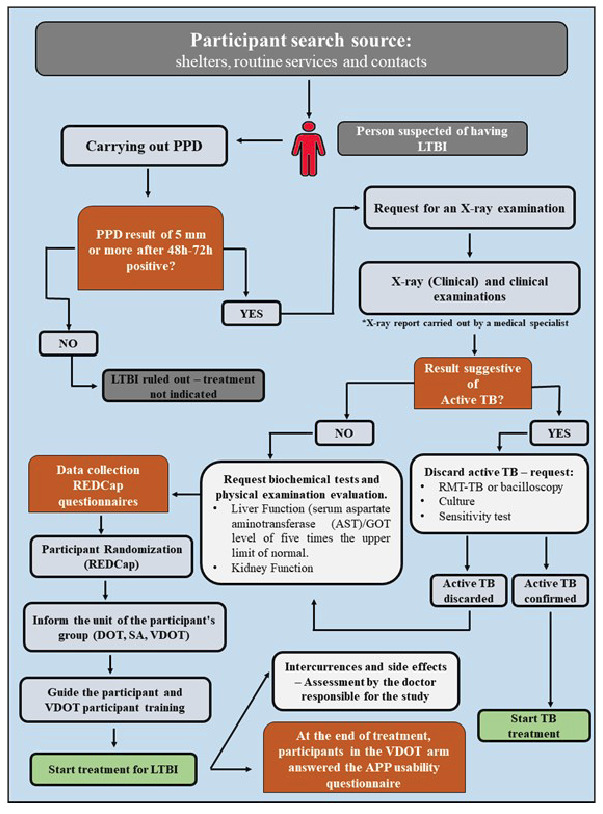
Study Flowchart.

Given the increase in the flow of international migrants to Manaus and the high prevalence of TB in this population, the implementation of short-course treatment (3HP) combined with VDOT (Figure 1) assists in the screening and treatment of this population. This therapeutic strategy reduces treatment duration, improves treatment adherence, and prevents the development of active and latent TB and its transmission within the community. Therefore, it is the responsibility of authorities and healthcare services to ensure equitable access to this treatment, safeguarding the health and rights of migrants, and contributing to the well-being of the entire Manaus population. Investing in the health of migrants is an investment in the future and the creation of a fairer and more inclusive society.
